# Market shaping via digital public infrastructure for assistive technology in low- and middle-income countries

**DOI:** 10.3389/fresc.2026.1881812

**Published:** 2026-09-01

**Authors:** Will Wade, Sijung Hu, James Fleming

**Affiliations:** 1 Research & Development, AACTools, Manchester, United Kingdom; 2 Clinical Research & Development Lead, Research & Development, Smartbox Assistive Technology, Bristol, UK; 3 Wolfson School of Mechanical, Electrical and Manufacturing Engineering, Loughborough University, Loughborough, United Kingdom

**Keywords:** assistive technology, data governance, digital public infrastructure, low- and middle-income countries, market shaping, offline-first

## Abstract

Digital assistive technology (AT) is now delivered through a variety of platforms, including smartphones, cloud services, dedicated devices, and embedded systems rather than a single dominant form factor. However, in low- and middle-income countries (LMICs), affordability, connectivity, electrical reliability, service capacity, and uneven language infrastructure continue to determine which digital pathways can be deployed and maintained at scale. This perspective argues that market-shaping strategies should prioritise the development of shared digital infrastructure beneath the application layer; this includes representative datasets and language resources, reusable models and reference toolchains, and deployment pathways tailored to resource-constrained environments. It proposes a three-part Market Stack comprising governed data and language resources, reusable core models and reference pipelines, and localised commercialisation and service delivery by small and medium enterprises. The central argument is not to replace smartphones but to ensure that digital AT markets in constrained settings work best when innovators and procurers can choose among smartphone, dedicated, embedded, and hybrid solutions. Therefore, funders and policymakers should view data, language resources, and foundational tooling as public-interest investments that widen the range of assistive products available in LMIC markets.

## 1 Introduction

The global unmet need for assistive technology (AT) is no longer contested. WHO-UNICEF reporting indicates that more than 2.5 billion people need at least one assistive product, with a major access gap concentrated in low- and middle-income countries (LMICs) (1). In this paper, LMICs denote economies that the World Bank classifies for analytical purposes as low-, lower-middle-, or upper-middle-income; it is an income-group shorthand, not a claim that countries across Africa, Asia, Latin America, or other regions share the same infrastructure, language, policy, or service conditions (2). Public summaries of the same evidence base note that access can fall as low as 3% in some settings (3). These figures have pushed governments, donors, and industry towards scale-oriented strategies, including digital channels.

Digital AT has genuine value in this context. Mobile devices can provide communication, reminders, navigation support, captioning, and interface adaptation without requiring custom hardware for every function, and AAC evidence from LMICs shows that digital and high-tech interventions can be effective when contextual support exists (4). Yet published work on assistive technology markets shows that affordability, service support, inclusive design maturity, and commercialisation barriers remain binding even when digital products exist (5–8). The practical question is therefore not whether digital delivery matters but which shared infrastructure makes digital AT reliable, adaptable, and commercially feasible in constrained settings.

This perspective argues that market shaping for digital AT in LMICs should focus on the layers below the application itself. It advances three linked claims: first, smartphone-first deployment is often useful but not sufficient because adoption depends on infrastructure readiness, workforce capacity, financing, and governance fit (9, 10); second, foundational data and language resources are a precondition for inclusive digital AT because accessibility datasets and low-resource language assets remain unevenly available (11–13); and third, reusable models, toolchains, and reference pathways are needed if local firms are to adapt these resources into maintained products (14, 15). The paper uses a digital public infrastructure lens to examine infrastructure fit, data representativeness, and the conditions under which assistive products can be translated into new language and service environments.

While this paper draws many examples from augmentative and alternative communication (AAC), reflecting the author’s field of practice, the same market-shaping logic applies across other digital AT domains, including vision, hearing, mobility, and cognitive support.

Digital AT markets are also diversifying across smartphone-native applications, dedicated assistive devices, embedded single-function products, and hybrid systems that combine local peripherals with cloud or edge services. This matters for market shaping because each pathway carries different requirements for power, connectivity, maintenance, and language support. An AAC function that is viable on a mainstream tablet in one setting may be delivered more robustly through a dedicated communicator, a phone-plus-peripheral model, or a low-power embedded pathway in another setting.

On that basis, we propose a “Market Stack” model for policy and implementation. The model treats key digital inputs as shared public-interest infrastructure, enabling competition to shift toward last-mile adaptation, service quality, and local relevance. In short, the Introduction establishes why a market-shaping argument is needed: digital AT access gaps persist because applications depend on infrastructure, language, data, affordability, and service conditions that no single product can resolve alone. The sections that follow therefore examine these conditions before presenting the Market Stack model.

## 2 Conceptual lens and method

### 2.1 Literature synthesis method

This perspective uses a narrative literature synthesis to develop a conceptual model, rather than reporting a systematic review or primary empirical study. Narrative synthesis is appropriate where the aim is to connect heterogeneous bodies of evidence and develop a conceptual argument across adjacent fields (16). The synthesis drew purposively on literature about assistive technology market shaping, AAC implementation in LMICs, digital health infrastructure, accessibility datasets, low-resource language resources, data governance, and digital public-interest infrastructure. These sources were read against three analytic questions: which infrastructure constraints shape the fit of different digital AT architectures; which data and language assets operate as shared market infrastructure; and which market functions are needed to translate upstream resources into localised products and services.

### 2.2 Digital public infrastructure lens

The digital public infrastructure (DPI) lens used here treats infrastructure as a shared, reusable, governed capability that enables many public, private, and civic actors to create downstream services. It is therefore not simply a claim about public ownership or digitised government services. Recent DPI scholarship argues that public value depends on the direction, governance, and common-good orientation of the infrastructure, not only on technical openness or scale (17). Applied to digital AT, this lens asks which data, language resources, models, toolchains, and reference pathways should be governed as public-interest enablers, as they expand the range of actors able to build affordable, localised, and maintainable products. The next section applies this lens first to infrastructure constraints and architecture fit.

## 3 Findings: infrastructure constraints and architecture fit

### 3.1 Infrastructure constraints change architecture fit

The smartphone-first paradigm is attractive because it starts from installed hardware and familiar user interfaces, and mobile or high-tech AAC interventions are already part of the LMIC evidence base (4, 18). In many settings it will remain the right default. But the policy problem is that “default” is often interpreted as “sufficient.” Evidence on digital AT and AAC in LMICs shows that barriers persist even when software exists, including device cost, training, repair, connectivity, and ongoing service support (4, 6, 8). High-tech AAC provision in LMICs already spans mainstream mobile devices, dedicated devices, and mixed arrangements rather than a single technical pathway (4).

Connectivity cost remains material for low-income users. International Telecommunication Union (ITU) affordability tracking shows progress globally but persistent inequity in low-income environments, including burdens that are substantially higher for the poorest households (19). A second issue is electrical reliability. WHO’s global analysis of electricity access in healthcare facilities reports continued gaps in reliable electricity, including only 40% reliability in sub-Saharan Africa (20). Household conditions are often more fragile than facility averages. In these conditions, uptime, charging, and recurring data cost become part of the assistive product itself rather than background assumptions.

This logic is consistent with broader LMIC digital health evidence: adoption outcomes are shaped by infrastructure readiness, workforce capacity, financing, and governance fit, rather than solely by the technical efficacy of an application (9, 10, 21, 22). The implication is segmentation, not smartphone rejection. Some use cases remain well served by phones, while others are better suited to lower power draw, offline operation, dedicated interfaces, or simpler maintenance.

### 3.2 Some AT functions merit alternative pathways

Current and emerging digital AT modalities already span several architectures. Smartphones remain important for flexible general-purpose access; dedicated AAC devices remain important where robustness, mounting, switch access, or specialised interfaces matter; and speech, gaze, and biosignal systems may rely on cloud or model-based components whose usefulness depends on local infrastructure and data availability. For many high-tech AAC systems, speech output is a core communicative function, so the availability of usable text-to-speech and related speech pipelines directly affects whether products can be localised and deployed in new language markets. For some narrowly scoped assistive tasks, a full operating-system stack may be unnecessary.

In practical market terms, at least four pathways are already visible: smartphone-native products; dedicated assistive devices; embedded or single-purpose products designed around one narrow function; and hybrid systems that pair commodity devices with external sensors, switches, mounts, speakers, or local peripherals. The significance of this taxonomy is not definitional. Each pathway implies a different profile of charging burden, repairability, accessory dependence, training need, and tolerance for intermittent connectivity. For LMIC market shaping, the relevant question is therefore not which pathway is most modern, but which one offers the best fit for a given assistive task under local infrastructure conditions (4, 8, 15).

Open-source hardware and software are already being used to create lower-cost AT tailored to specific functions (15). Embedded speech work likewise shows that narrow voice functions can now run on constrained devices (23, 24). These examples do not justify a blanket ban on smartphones. They support a narrower policy claim: procurement and design processes should test architecture fit function by function, including offline-capable and purpose-built hardware where general-purpose stacks are fragile or unnecessarily costly.

This section supports the paper’s main argument by showing that digital delivery channels are not interchangeable: infrastructure conditions shape which architectures can provide reliable access. The next section moves upstream from device and deployment fit to the data and language resources that determine whether digital AT can be localised at all.

## 4 Findings: data and language resources as market infrastructure

### 4.1 Biosignal representation gaps

As digital AT moves toward personalised interaction and machine-learning-enabled support, data quality and representativeness become central constraints. This is not only a scale problem (how much data exists) but also a fit problem (whether the data reflect target users and deployment conditions).

Eye-gaze systems are a concrete example of this fit problem: models that perform well in controlled cohorts may not transfer cleanly to intended clinical populations.

For example, studies of eye-gaze-mediated access in children with dyskinetic cerebral palsy report performance variability and practical service challenges, while the eye-gaze service literature emphasises individualised assessment, support networks, follow-up, and funding processes as determinants of successful use (25–27). Clinical populations may also exhibit ocular or motor characteristics that differ from the convenient cohorts often used in technical validation studies. Models validated on convenient cohorts can therefore underperform in intended assistive cohorts even when algorithmic performance appears strong in laboratory conditions.

### 4.2 Representative disability data remain scarce

The challenge is broader than any single modality. A recent meta-analysis of accessibility datasets reports weak reporting and limited representativeness across key demographic dimensions, including gaps in the documentation of race/ethnicity and gender (11). IncluSet further documents that many accessibility datasets are difficult to discover or not openly shared, often gated by “available on request” workflows that impede reproducibility and product translation (12). For brain–computer interface (BCI) development, reviews show a related pattern: many public datasets are still concentrated in paradigms recorded from healthy participants, while AAC-BCI evidence for people with disabilities remains heterogeneous, small-sample, and inconsistently reported (28, 29). This does not invalidate existing work, but it does limit generalizability to populations that are central to rehabilitation practice.

### 4.3 Linguistic primitives as infrastructure, not optional extras

Speech-related AT is often discussed as if model architecture is the limiting factor. In many LMIC language settings, core bottlenecks are earlier: phonemisation resources, phonological mappings, morphological resources, aligned speech corpora, and pronunciation lexicons. Recent low-resource NLP evidence shows that phonemic representations can improve the robustness of zero-shot cross-lingual transfer, including for non-Latin scripts (30). This supports treating phonemisation resources as foundational language infrastructure.

Work on East African languages shows that building usable text and speech datasets, benchmarks, and models is itself a major infrastructural task for low-resourced settings rather than a downstream packaging step (13, 31). Mozilla Common Voice provides a concrete example of this infrastructural logic: an open, crowdsourced speech corpus designed as reusable multilingual infrastructure rather than for any single downstream product, with subsequent localisation work showing how such shared platforms can help extend speech-resource development into under-resourced language communities (32, 33). The uneven distribution of language resources across the NLP ecosystem therefore creates a public-interest case for funding pre-model linguistic infrastructure, not only later-stage AI systems (14). For firms seeking to enter new language markets, the gating issue is often not interface design but whether usable text-to-speech and related language assets exist at all. Restrictive licensing can intensify this bottleneck by making downstream integration and maintenance harder. Public-interest actors such as ministries, universities, disability organisations, and mission-driven funders should therefore treat these resources as enabling infrastructure rather than waiting for downstream vendors to solve the problem on their own.

This is also a practical constraint for established AAC suppliers, including in the author’s own industry context. To launch in a new language market, a supplier may need far more than translated labels: text normalisation, pronunciation resources, localised vocabularies and symbols, speech voices that sound acceptable to users, and a deployment path that works on the relevant class of hardware. Where those prerequisites are absent, localisation becomes bespoke development with uncertain maintenance costs. Shared language assets and reusable speech pipelines can therefore lower entry barriers for incumbent AT firms and local small and medium enterprises (SMEs) alike by turning repeated localisation effort into governed, reusable infrastructure (13, 14, 34).

Taken together, these findings justify treating data and language resources as market infrastructure rather than as downstream product features. The discussion now converts that synthesis into the Market Stack model.

## 5 Discussion: the market stack for digital AT

The three layers were developed by identifying recurring patterns in the literature on what digital AT markets need to work. The layers are not yet a tested framework; they are a way to organise the main functions that appear across the evidence. Layer 1 captures upstream resources that the literature identifies as necessary for inclusion but unlikely to be supplied adequately by individual firms alone. Layer 2 captures the technical conversion function: turning those resources into reusable models, tools, and reference pathways. Layer 3 captures the last-mile function of adapting, financing, delivering, and supporting products in specific local markets. The digital public infrastructure lens shaped the classification by asking whether an input is shared, reusable, governance-dependent, and enabling of downstream participation.

**Figure 1:**
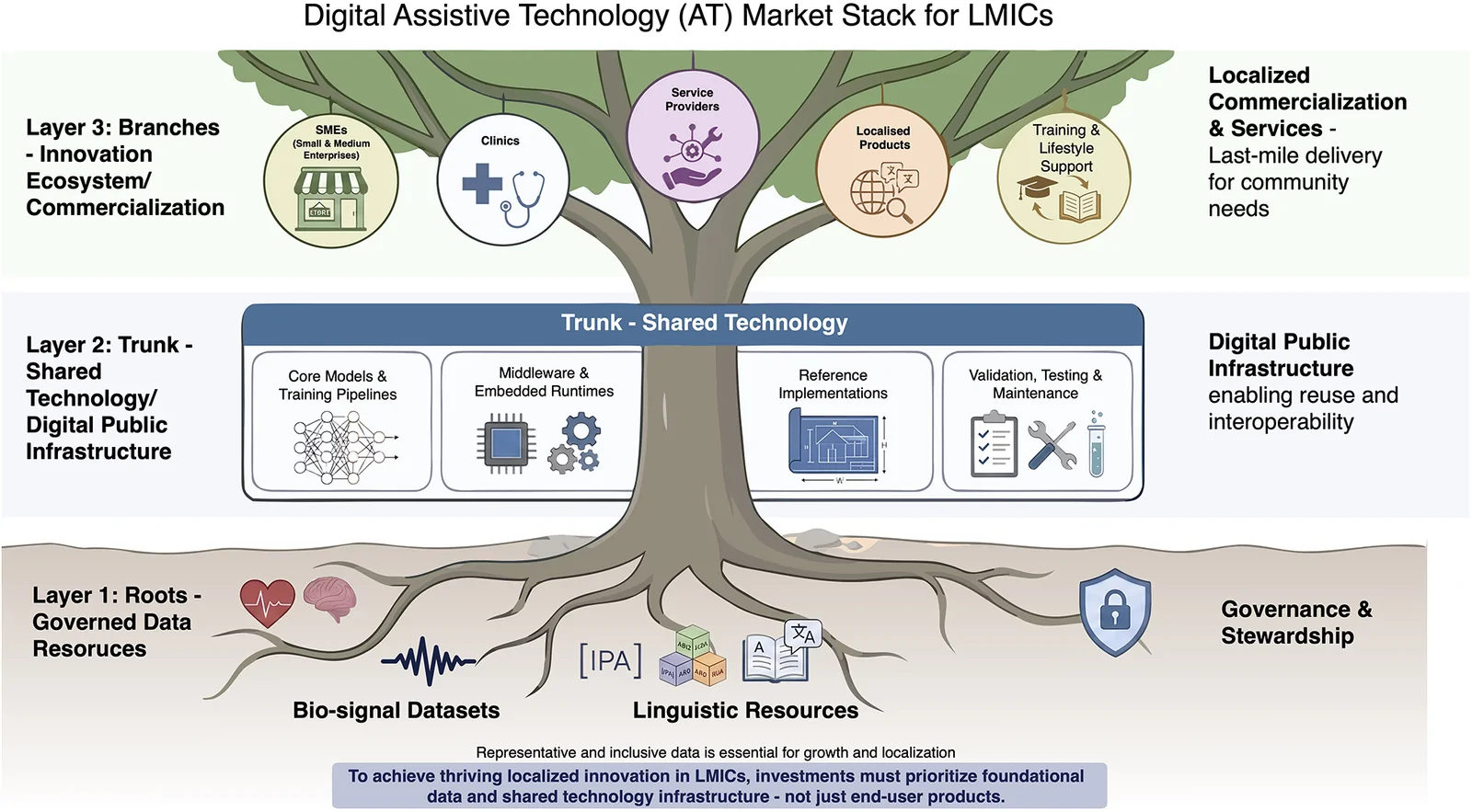
Market Stack tree for digital assistive technology. The roots (Layer 1) provide governed foundational data and primitives, the trunk (Layer 2) concentrates these inputs into core models and toolchains, and the branches (Layer 3) represent localised commercialisation, products, and service delivery.

The market-shaping implication is to treat shared digital inputs as infrastructure rather than expecting every supplier to recreate them independently. The tree metaphor used in Figure 1 clarifies dependency flow and market function: roots correspond to public-interest infrastructure that is hard for individual firms to finance alone; the trunk corresponds to shared technical pathways that convert these assets into reusable capability; and branches correspond to the commercial and clinical endpoints where localised products, services, and training are delivered.

### 5.1 Pre-stack equity conditions

The Stack does not assume that supply-side infrastructure automatically solves distributive justice problems. In LMIC settings, people with disabilities may face prior barriers of affordability, digital literacy, transport, stigma, weak entitlement systems, limited political voice, and uneven disability-service capacity (18, 35–37). Rather than adding a literal “Layer 0” to the figure, the model treats these issues as pre-stack equity conditions that cut across all three layers. If they are ignored, shared datasets or toolchains may still reproduce exclusion because disadvantaged users cannot afford devices, participate in design decisions, obtain repairs, or influence procurement.

The outcome variable is therefore not infrastructure creation in itself. It is sustained equitable access: affordable acquisition and use, local-language usability, reliable support and repair, participation of people with disabilities and their organisations in governance, and opportunities for local providers to deliver services. Equity sits on both sides of the Stack: upstream governance must include representative users and communities, while downstream market activation must ensure that benefits reach people who are otherwise least likely to be served.

### 5.2 Layer 1: governed data and language resources

Layer 1 is the foundation: investment in datasets and linguistic assets that the market undersupplies but are essential for inclusive digital AT. For sensitive human data, the model is not for unrestricted public release. It is open metadata, with governed access and explicit stewardship rules.

FAIR principles provide technical guidance for findability, interoperability, and reuse (38). CARE principles provide governance guidance for collective benefit, authority, responsibility, and ethics, especially where community data rights are salient (39). Data stewardship mechanisms such as trusts, cooperatives, and contractual access frameworks provide implementable options, each with trade-offs in accountability and administrative overhead (40).

For speech-related AT specifically, Layer 1 resources include phonemisation lexicons, pronunciation mappings, timestamped aligned transcription corpora, and representative voice datasets. These are the preconditions for viable local text-to-speech (TTS) and speech pipelines. For biosignal AT, Layer 1 includes representative cohorts and task metadata required for transfer into intended clinical populations.

### 5.3 Layer 2: foundational tooling, middleware, and reference pathways

Layer 2 covers reusable software and hardware components built on Layer 1 resources, including core models, training pipelines, and reference implementations that local firms can integrate without re-creating common infrastructure. In practical terms, this is the layer where datasets become reusable speech engines, middleware, embedded runtimes, and reference designs that downstream suppliers can actually deploy. Licensing is best understood here as an enabling condition rather than a separate layer: where the policy objective is broad local integration and downstream competition, reusable terms can reduce transaction friction while preserving attribution, safety, and accountability requirements.

In text-to-speech terms, Layer 2 includes reusable models and toolchains trained on Layer 1 primitives; these can then be modified, improved, and productised in local deployments under clear downstream-use terms. This layer can also include speech engines, language tooling, embedded inference runtimes, validation datasets, and reference electronics for narrow AT functions. The practical value is market entry: if shared TTS and language tooling exist, specialised AT suppliers can adapt products for new language markets without rebuilding the entire speech stack from scratch.

Layer 2 is also where commercialisation becomes technically tractable. An established AAC supplier may already have mature user interfaces, access methods, and clinical workflows, yet still be unable to enter a new language market if it must separately build a phonemiser, speech model, embedded runtime, test corpus, and hardware integration path from first principles. The same constraint is even sharper for smaller local firms. By contrast, maintained reference implementations and deployable runtimes shorten the path from language resource to usable product. Recent edge-speech work suggests that narrow speech functions can be deployed on constrained hardware when models and toolchains are designed for local inference rather than assumed cloud dependence (23, 24). The policy implication is not that every AT product should run on a microcontroller but that Layer 2 should preserve architectural choice by making smartphone, dedicated-device, and edge pathways all technically reachable.

### 5.4 Layer 3: localised last-mile commercialisation and service

Layer 3 is where local enterprises, clinics, and service providers adapt shared building blocks into context-specific products and support pathways. This is the primary commercialisation layer. When Layers 1 and 2 are available under clear, reusable terms, firms can compete on last-mile value: language adaptation, cultural representation, device form factor, power profile, training, repair, and service quality. For that to happen, procurement and reimbursement rules must permit qualified local entrants to participate, and post-deployment support must be budgeted rather than assumed. To cross the commercialisation valley of death, Layer 3 also needs market-activation finance, including pooled procurement, advance market commitments, and matched grant programmes for local manufacturers and service partners (6, 8, 41, 42).

Figure 1 summarises the conceptual contribution after these layer definitions: upstream resources are governed as roots, reusable technical pathways form the trunk, and localised products and services emerge as branches.

### 5.5 Layer interactions and empirical next steps

The Stack should be read as an interactive system rather than a fixed one-way hierarchy. Upstream resources enable downstream products, but downstream experience should also reshape upstream priorities. For example, Layer 3 service failures can reveal that a Layer 2 reference implementation is too connectivity-dependent, that a Layer 1 speech corpus misses an important accent or disability group, or that procurement rules exclude local repair providers. Similarly, local user feedback, clinical practice, and SME experience should inform dataset governance, validation criteria, and reference-tool design.

This feedback matters because many LMIC settings lack robust systems for data stewardship, language resource development, local validation, and lifecycle support. Trust and technology transfer therefore require explicit arrangements: participatory governance with people with disabilities and local organisations, transparent data-access rules, local validation before procurement, training and maintenance contracts, and knowledge-transfer obligations for global developers working with local partners (17, 39, 41). Without those mechanisms, the Stack could become another upstream supply initiative with weak accountability to intended users.

The model is therefore a conceptual starting point, not a validation-ready framework. Empirical next steps should include product-category case studies, country-level readiness assessments across all three layers, and pilot investments that compare outcomes before and after shared infrastructure is introduced. Relevant test indicators include total cost of ownership, uptime, repair turnaround, local-language coverage, user satisfaction, participation of people with disabilities in governance, and the number and durability of local suppliers able to deliver supported products.

The model is analogous to other digital public-good ecosystems in which public-interest actors maintain reusable infrastructure while commercial actors compete in integration, deployment, and support. In this structure, market differentiation occurs mainly in adaptation, maintenance, and service quality rather than ownership of foundational components.

### 5.6 Infrastructure enablement and justice

The equity argument in this paper is therefore deliberately limited. It does not claim that infrastructure enablement replaces distributive justice, social protection, subsidy design, rights enforcement, or anti-discrimination policy. Those remain necessary for people with disabilities to obtain affordable AT in practice (35, 36). The claim is that distributive measures cannot fully succeed if the digital supply base is missing: if there are no representative datasets, usable local-language resources, deployable reference tools, or serviceable technical pathways, even well-funded procurement may have little to buy that is affordable, localised, and maintainable. The Market Stack therefore situates equity and access through infrastructure enablement as a necessary but insufficient complement to justice-oriented financing and entitlement policies.

## 6 Policy recommendations

The Market Stack points to five practical recommendations for funders, governments, procurers, and implementation partners.

First, country- and product-category-level assessments should map readiness across all three layers before procurement or scale-up decisions are made. A digital AT opportunity should be assessed not only for product efficacy, but also for electricity, connectivity, language coverage, representative data, local validation capacity, repair pathways, and long-term service financing (6, 8, 9).

Second, funders should finance Layer 1 resources as public-interest infrastructure. This includes governed disability datasets, speech and language corpora, pronunciation resources, open metadata, and data-stewardship arrangements that apply FAIR and CARE principles while protecting sensitive data (12, 38, 39).

Third, Layer 2 investments should produce reusable reference tools rather than one-off pilots. Priority outputs include language toolchains, speech pipelines, embedded and offline-capable runtimes, validation datasets, and reference implementations with licensing terms that allow qualified local firms to adapt and maintain products.

Fourth, Layer 3 market activation should be built into procurement and reimbursement. Governments and donors should use pooled procurement, advance commitments, matched grants, and service contracts to help local SMEs, clinics, and repair providers participate in delivery, rather than reserving market access only for established international suppliers (6, 41, 42).

Fifth, every layer should include equity safeguards. People with disabilities and their organisations should participate in governance and validation; affordability should be addressed through subsidies or social protection mechanisms; and monitoring should track sustained access outcomes such as total cost of ownership, uptime, repair turnaround, language usability, and user satisfaction (35, 36).

These recommendations translate the conceptual model into implementation choices while leaving space for empirical testing and local adaptation.

## 7 Conclusion and future work

This perspective argues that digital AT access in LMICs is shaped not only by the availability of applications but also by the fit between the technical architecture and local infrastructure. Smartphone-first delivery will often remain appropriate, but it should not be treated as the only pathway. Where power, connectivity, repair capacity, language coverage, or interface needs are limiting, dedicated, embedded, and hybrid designs may offer more reliable access. The practical policy question is therefore which architecture best fits the assistive function and service environment.

The same principle applies upstream. If representative disability data, language resources, and reusable speech or biosignal pipelines are missing, local adaptation becomes slow, expensive, and fragile. The Market Stack is intended to clarify this dependency: Layer 1 resources provide the foundation for Layer 2 tooling and reference pathways, which in turn enable Layer 3 firms and service providers to compete on localisation, training, repair, and support rather than rebuilding core infrastructure. For funders and policymakers, the implication is to treat shared datasets, linguistic assets, and foundational tooling as public-interest investments and to align procurement with offline use, local-language support, and lifecycle service requirements (6, 8, 38–42).

This paper is intentionally conceptual and draws heavily on AAC examples. Future work should test the Market Stack across specific product categories and country cases and examine whether shared infrastructure investments improve service continuity, total cost of ownership, repair turnaround, and local market participation. Even so, the central conclusion is clear: market shaping for digital AT in LMICs should widen the set of technically and commercially viable pathways, rather than assume that one device model or one software channel will fit every context.
